# A cluster-randomised controlled trial to promote physical activity in adolescents: the Raising Awareness of Physical Activity (RAW-PA) Study

**DOI:** 10.1186/s12889-016-3945-5

**Published:** 2017-01-04

**Authors:** Nicola D. Ridgers, Anna Timperio, Helen Brown, Kylie Ball, Susie Macfarlane, Samuel K. Lai, Kara Richards, Winsfred Ngan, Jo Salmon

**Affiliations:** 1 Institute for Physical Activity and Nutrition (IPAN), School of Exercise and Nutrition Sciences, Deakin University, 221 Burwood Highway, Burwood, VIC 3125 Australia; 2 School of Exercise and Nutrition Sciences, Deakin University, Melbourne, Australia

**Keywords:** Online, Leisure time, Sedentary behaviour, Wearable technology, Pedometer

## Abstract

**Background:**

Recent technological advances provide an alternative yet underutilised opportunity for promoting physical activity in youth. The primary aim of the Raising Awareness of Physical Activity (RAW-PA) Study is to examine the short- and longer-term impact of a wearable activity monitor combined with digital behaviour change resources on adolescents’ daily physical activity levels.

**Methods/Design:**

RAW-PA is a 12 week, multicomponent physical activity intervention that utilises a popular activity tracker (Fitbit® Flex) and supporting digital materials that will be delivered online via social media. The resources target key behaviour change techniques. The intervention structure and components have been informed by participatory research principles. RAW-PA will be evaluated using a cluster randomised controlled trial design with schools as the unit of randomisation. Twelve schools located in Melbourne, Australia, will allocated to either the intervention or wait-list control group. The target sample size is 300 Year 8 adolescents (aged 13–14 years). Participants’ moderate- to vigorous-intensity physical activity will be the primary outcome. Survey measures will be completed. Process factors (e.g. feasibility, acceptability/appeal, fidelity) will also be collected.

**Discussion:**

To our knowledge, this study will provide some of the first evidence concerning the effect of wearable activity trackers and digital behaviour change resources on adolescents’ physical activity levels. This study will provide insights into the use of such technologies for physical activity promotion, which may have a significant impact on health education, promotion, practice and policy.

**Trial registration:**

Australian and New Zealand Clinical Trials Registry No: ACTRN12616000899448. Date of registration: July 7, 2016.

## Background

Regular physical activity benefits youth physical, social, mental and emotional health, including psychological well-being, bone health and fitness [1]. In contrast, low levels of physical activity are associated with the increased likelihood of cardiovascular disease risk factors including metabolic syndrome, higher waist circumference, and overweight/obesity [2]. However, only 13% of Australian 12–14 year olds [3] engage in 60 min of moderate- to vigorous-intensity physical activity (MVPA) every day - the current recommendation for health [4]. Low guideline compliance has also been observed in other developed countries [5–7]. Adolescence is an age where declines in physical activity levels are common [8]. This is of particular concern as this life-stage represents a time where health inequities start to emerge, and these may extend into adulthood [9]. For example, those living in socioeconomically disadvantaged areas are at a greater risk of declines in their activity levels [10] and are less likely to meet national activity guidelines [11]. Consequently, primary preventative measures targeting adolescents, particularly among those living in disadvantage, are warranted.

The majority of physical activity interventions in young people have targeted primary school children, with fewer initiatives designed specifically to increase activity levels in adolescents [12, 13]. There is also a lack of intervention studies that have specifically targeted the promotion of activity levels of adolescents living in disadvantaged neighbourhoods [14]. Of those conducted, a number targeted a single sex [15–18] and most have used multicomponent approaches delivered through school-based settings [15, 16, 19–21]. Such interventions can be resource-intensive, costly and are usually conducted in class, which can be difficult to implement due to an already crowded curriculum. All of these factors have a negative impact on the reach and sustainability of such approaches. In addition, existing approaches have often focused on sport [13, 20, 21], which may not be appealing to inactive adolescents who have little or no involvement in organised sport [22]. As such, there is a need for further research to examine non-curriculum based, lower resource intensive approaches for promoting physical activity levels in adolescents living in socioeconomically disadvantaged areas.

Recent technological advances provide an alternative, yet underutilised opportunity for promoting physical activity in youth. Wearable activity trackers (e.g. Fitbit®, Garmin®, etc.) are self-monitoring tools that have the capacity to track physical activity in real-time and provide individualised feedback against set goals and physical activity recommendations. They are accompanied by apps and/or web-based portals that incorporate a range of behaviour change techniques, including social support, prompts/cues, biofeedback, and focus on past successes [23, 24]. Notably, such technologies have considerable mass market appeal, are increasingly popular, and are being widely adopted across all age and socioeconomic groups [25]. For example, in the US, one in 10 adults own an activity tracker and ~60% continue to use it after 12 months [26]. In Australia, 20% of adults own a wearable activity tracker [25]. Whilst there are no data available concerning adolescent ownership or use of activity trackers, these technologies are likely to have substantial appeal to youth as they are often early adopters of new technologies [27]. Despite this, little research has examined whether these devices can be effectively utilised to increase physical activity among adolescents [28]. The majority of interventions using such devices have focused on adults, with mixed evidence concerning the efficacy of wearable devices for increasing overall activity levels [24, 29, 30].

Some research has suggested that wearable activity trackers can motivate individuals to make enduring changes to their daily activity [31]. However, several recent studies conducted with adults have questioned the value of such devices for promoting physical activity levels, suggesting that self-monitoring alone may not be sufficient to increase activity levels and that additional support may be required to help change behaviour [29, 32]. Online programs (e.g., web-based programs providing social support, tailored programs), social media platforms (e.g., Facebook groups), and digital resources such as videos, images, and infographics may help to overcome this limitation and educate and provide individuals with behaviour change techniques and skills. In addition, online programs have significant advantages in that they are able to reach a large target audience, are readily accessible, and use social connections and networks to engage and motivate participants [33]. Since recent data suggest that 96% of adolescents in this target age group have home internet access [34], and Facebook is the most popular and frequently used social media platform [35], integrating wearable activity trackers with digital behaviour change resources hosted online on social media platforms may be one intervention strategy that can positively influence physical activity levels in this target group.

This paper provides a rationale and description of the Raising Awareness of Physical Activity (RAW-PA) Study protocol; an innovative physical activity intervention that combines wearable activity trackers with online digital behaviour change resources for inactive adolescents attending schools in socioeconomically disadvantaged areas.

### Aims

The primary aim of RAW-PA is to examine the short- and longer-term impact of a wearable activity tracker combined with behaviour change resources on adolescents' daily MVPA. In order to understand patterns of change in activity levels (e.g., How do activity intensities change? When during the day do changes occur?), this study will evaluate the short- and longer-term impact on sitting time and MVPA across the whole day and during periods of the day (e.g., school hours). In addition, given the scarcity of evidence with this target population, this study will evaluate the impact of the intervention on potential mediators (e.g. self-efficacy, social support, etc.) to examine how the intervention effected change. Lastly, this study will examine process factors (feasibility, acceptability/appeal, fidelity).

## Methods

### Study design overview

RAW-PA will be evaluated using a cluster randomised controlled trial (cluster-RCT) design with secondary schools being the unit of randomisation. The intervention will target adolescents in Year 8 (second year of secondary school) in 12 co-educational, government funded schools in Melbourne, Australia. The intervention will run for 12 weeks, with assessments to be conducted at baseline, post-intervention and at 6-month follow-up (see Table 1 for the proposed study timeline). The design, conduct and reporting of this RCT will adhere to the Consolidated Standards of Reporting Trials (CONSORT) guidelines. Ethical approval was obtained from Deakin University Human Research Ethics Committee (2016–179) and the Victorian Department of Education and Training. The trial is registered with the Australian and New Zealand Clinical Trials Registry (ANZCTR): ACTRN12616000899448.

**Table 1:**
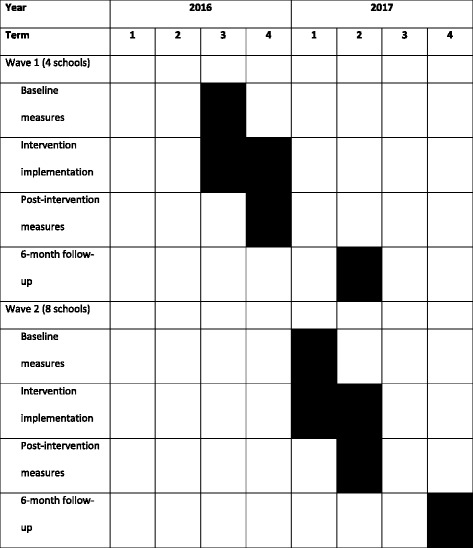
Proposed timeline of RAW-PA Study

### Settings and participants

The Socio-Economic Index for Areas (SEIFA [36]) will be used to identify socioeconomically disadvantaged suburbs (SEIFA index of ≤5) in Victoria, Australia. SEIFA summarises the characteristics of people and households within an area and uses a number of criteria, including employment, education, housing stress and family type [36]. The My School website (www.myschool.edu.au) will then be used to identify schools located in socioeconomically disadvantaged suburbs that are located within ~60 km of Deakin University’s Burwood Campus. Eligible schools will be randomly selected and invited to participate in the study until 12 schools agree. Eligible study participants will be adolescent males and females who are in Year 8, are at least 13 years old (minimum age required to have a Fitbit® and a Facebook account) and attend one of the recruited schools. Eligibility criteria include: (1) having access to the Internet outside of school (via smartphone or home Internet); (2) having (or willing to create) a Facebook account; (3) not engaging in regular organised physical activity/sport outside of school (at least once per week throughout the year); (4) not meeting national physical activity guidelines of at least 60 min of MVPA every day; and (5) not a current or past owner of an activity tracker. Eligibility will be determined based on a checklist completed by parents and students. The first 25 eligible students from each school to return a completed informed written parental consent form (which includes student assent) will be recruited into the study.

### Sample size

Previous population-based studies were used to estimate the SD of mean daily minutes of MVPA (18.6 [37]) and the intraclass correlation coefficient for clustering within schools (ICC; 0.01 [38]). Based on an initial sample size of 300 adolescents (150 per study arm) participating from 12 schools (25 students per school), the z-statistic for 80% power is 0.84 and the z-statistic for α = 0.05 is 1.96. Based on 70% providing usable data at post-intervention, it was estimated that there would be 210 students (105 per arm) at post-intervention. With this sample size, the study is able to detect a difference in the mean daily MVPA between intervention and control students of 7.9 min at post-test.

### Blinding and randomisation

In this cluster-RCT, recruitment and baseline data collection will be conducted prior to randomisation. Schools will be match-paired based on their size and SEIFA score and randomly allocated to either the intervention (6 schools) or wait-list control (6 schools) group by a computer-based random number generator. Randomisation will be conducted by an independent researcher not involved in the current study. Participants attending schools randomised to the intervention group will commence the program once baseline measures have been completed for all participants. Participants in the wait-list control group will be provided access to the intervention materials after the completion of the 6-month follow-up assessments.

### Intervention

RAW-PA is a 12-week multicomponent physical activity intervention that utilises a widely available and popular activity tracker to target physical activity levels, with supporting digital materials that have been designed to target evidence-based behaviour change strategies. The intervention has been informed by our pilot research (conducted August-December 2015; see ‘Development of RAW-PA’ below) that used participatory research principles involving adolescents in designing and reviewing the intervention structure and components. This approach has been used to ensure that the intervention is tailored to adolescents’ needs, which is important for instilling feelings of ownership and control [39]. The intervention consists of the following components: (a) wrist-worn Fitbit® Flex; (b) accompanying Fitbit® app (free to register and use); (c) interactive weekly individual and/or team ‘missions’ or ‘challenges’; (d) digital behaviour change resources including infographics, short informative and motivational videos and social forums, accessible via a private, proactively researcher-moderated Facebook page; and (e) alerts to new content and missions or challenges delivered via email and/ or text message ~2–3 times/week. The accompanying behaviour change resources match the theme of the weekly ‘mission’ and are designed to step participants through the behaviour change process in a flexible, interactive way. They are based on behaviour change techniques (described in more detail in the following sections) that are effective for changing behaviour at the individual and population levels [40]. The online delivery mode ensures that the digital resources are readily accessible for adolescents to engage with in their own time using computers or mobile devices. The intervention has been designed to target low-cost everyday physical activity (e.g. walking with friends, active transport) that can be integrated into adolescents’ daily lives. An overview of the weekly ‘missions’ is provided in Table 2.

### Theoretical basis of RAW-PA

Previous research has demonstrated that multicomponent [41], short-term technology-based interventions [33] can effectively increase adolescents’ physical activity levels. Interventions based on behavioural theories are also more likely to be effective than atheoretical approaches [42]. RAW-PA is grounded in Social Cognitive Theory [43] and Behavioural Choice Theory [44]. These theories recognise that health behaviours are influenced by factors operating at multiple levels including intrapersonal (e.g. enjoyment, self-efficacy) and interpersonal (e.g. families, teachers) influences. The core component of RAW-PA is the Fitbit® Flex, which incorporates 20 behaviour change techniques within the monitor and the accompanying app [23]. The additional RAW-PA interactive weekly ‘missions’ or ‘challenges’ and accompanying resources are designed to help students learn and develop key behaviour change techniques (e.g. self-monitoring, goal setting, social support, self-efficacy, action planning etc.) that are recognised as being critical for having the confidence to change and maintain changes in behaviour [40]. RAW-PA targets the accumulation of activity (steps) throughout the day and strategies for how to integrate more movement — typically walking — into daily life. Activity accumulation may be more appealing for inactive adolescents who may be disengaged from sport or higher intensity activities. An overview of the intervention approach, the targeted determinants, and the behaviour change techniques targeted by the weekly ‘missions’ is provided in Table 2.

**Table 2:** Description of RAW-PA objectives, theoretical approach and behaviour change techniques targeted

Week	Theme	Intervention objective	Determinants or mediators^a^	Behaviour change techniques^b^[40]
1	Knowing is the First Step!	Familiarisation with the Fitbit® Flex	Knowledge	Self-monitoring behaviour
2	Build it Up!	Developing goal setting skills Identifying barriers to physical activity engagement	Self-efficacy Benefits/barriers	Goal-setting (behaviours) Problem solving
3	Pair Up!	Encourage friends/peers to increase their activity levels	Modelling behaviour Social support	Social support - practical Social support - emotional
4	The Happy Dance	Celebrating the achievement of set goals	Self-efficacy Benefits/barriers	Goal-setting (behaviours) Action planning Social reward
5	It’s a Social Movement	Providing social support for physical activity	Enjoyment Outcome expectations	Social support - practical Social support - emotional
6	Reach for the Stars!	Tracking activity levels across the day in comparison to sports stars	Enjoyment Self-monitoring and contracting	Problem solving Instruction on performing a behaviour Social comparison
7	Break it Up!	Identifying and sharing strategies for breaking up sitting time	Self-efficacy Modelling behavioural	Habit reversal Self-efficacy Social support - practical
8	Step it Up!	Evaluating and adjusting set goals	Self-efficacy Barriers/benefits	Focus on past success Review of outcome goals Set graded tasks
9	Buddy Up!	Support friends/family to increase their own physical activity	Modelling behaviour Enjoyment	Modelling behaviour Identification of self as role model
10	Mark it Up!	Identifying and sharing strategies for increasing steps at school Raising awareness of activity opportunities provided at school	Availability Access	Social support Social comparison Action planning
11	Globe Trotter	Evaluating set goals Reflecting on journey so far	Self-efficacy Outcome evaluations	Self-monitoring Review of outcome goals
12	Keep it Up!	Message reinforcement Confidence to be physically active	Knowledge Self-efficacy Benefits/barriers	Commitment Review of outcome and behavioural goals Relapse prevention

^a^Based on Social Cognitive Theory [43] and Behavioural Choice Theory [44]

^b^Behaviour change techniques from the Behaviour Change Technique taxonomy [40]

### Development of RAW-PA

To inform the development of the current intervention using participatory research principles, the research team piloted the feasibility, usability and acceptability of the Fitbit® Flex and accompanying app/web-based portal in 60 Year 8 adolescents (aged 13–14 years; 100% response rate) from three secondary schools (one low, one mid and one high socio-economic status (SES)) in Melbourne. As there is a dearth of information concerning the feasibility of these devices in adolescents [28], exploring the experiences of adolescents from different SES areas provided insights into whether these technologies offer promise for promoting physical activity levels among adolescents with a range of socioeconomic circumstances.

The Fitbit® was selected as it accounts for a large proportion of activity tracker sales [45], performs well in comparison to research grade monitors [46], and the accompanying app is free to use. Students wore the Fitbit® Flex for six weeks and completed three surveys at the end of Weeks 1, 3 and 5. After Week 6, students were interviewed regarding their thoughts about activity trackers and how to integrate such technologies into a physical activity intervention. Adolescents were asked to provide feedback about how the program should be delivered (e.g. style, frequency), the format and content of the digital (internet-based) resources, and strategies for facilitating engagement and motivation during the intervention was sought. Adolescents generally reported that the Fitbit® was easy to use (97% agreed at Week 1, 100% agreed at Weeks 3 and 5), over 80% wore the Fitbit® on any given day, the Fitbit® was used regularly to track daily activity (over 70% at each time point checked their activity ≥2 times a day), and their awareness of their activity levels (≥90% week 1) and intentions to be more active increased (≥80% at week 1) as a result of the Fitbit®. However, adolescents also indicated that wearing an activity tracker (alone) may not be enough to increase activity levels (≥40% agreed in week 1, ≥50% week 5). RAW-PA was subsequently developed based on the adolescents’ feedback, and suggestions about the content and presentation of the resources were reviewed by the adolescents and refined further.

### Measures

Research assistants will conduct all student assessments in schools. Parents will complete surveys at home. To ensure consistency between research assistants, a protocols document has been developed for use at all data collections and all research staff will undergo a training session prior to assessments. A range of measures are described below and will be collected at baseline, post-intervention and at 6-months post-intervention, unless otherwise stated.

### Physical activity and sedentary time

#### Accelerometry

Physical activity and sedentary time will be objectively-assessed using hip-mounted ActiGraph accelerometers (model GT3X+; ActiGraph, Pensacola, FL, USA). Students will be instructed to wear the accelerometer for 8 consecutive days at each time point during waking hours (except during water-based activities). The ActiGraph is the most commonly used accelerometer in youth research [47] and has acceptable validity and reliability for assessing adolescents’ free-living activity levels [48]. Raw acceleration data will be sampled, downloaded and processed into 15 s epochs using manufacturer proprietary software. Age-specific thresholds will be used to determine time spent in moderate— and vigorous-intensity physical activity [49]. Moderate— and vigorous-intensity physical activity will be summed to determine time spent in daily MVPA. Sedentary time will be defined as ≤100 cpm [50]. Time spent sedentary or physically active for the whole day and specific periods of the day (e.g. after school) will be obtained.

#### Survey measure

Activity levels will be assessed using a brief self-report measure that asks adolescents to report the number of days (0–7) they were physically active for a total of at least 60 min/day (1) over the past 7 days and (2) over a typical or usual week [51]. Responses to these items will be averaged for use in the analyses. This measure has been validated for use with Australian adolescents [52].

#### Leisure-time physical activity and sedentary behaviours

Adolescents’ leisure-time behaviours will be assessed using items adapted from the validated Middle-School Physical Activity and Nutrition (M-SPAN) Survey [53]. Students will be asked how much time they spend in leisure-time sedentary behaviours such as watching television, using the internet and doing homework, using a 6-point scale ranging from none to 4+ hours/day on weekdays and weekends. Usual mode of transport to and from school will also be assessed using an adapted measure from a survey developed by Timperio and colleagues [54]. Students will be asked to report the main method of transport (i.e., walked, cycled, car, public transport) to school and from school each weekday.

### Mediators of behaviour change

As noted above, this study will also examine potential mediators of behaviour change. Few interventions examine potential mediators, even those based on behaviour change theory, despite the potential for such information to provide insights into why an intervention may or may not be efficacious [55]. The following potential mediators of behaviour change will be examined as they have been examined previously in children [56, 57] and adolescents [58, 59], and they are being specifically targeted through the 12-week program via the weekly missions (see Table 2).

#### Self-efficacy

Self-efficacy will be assessed using five items from an existing scale that has acceptable validity and reliability in young adolescents [60]. Students will respond to each question using a 6-point Likert-type scale, with responses ranging from disagree a lot (1) to agree a lot (6).

#### Social support

Social support from friends and family will be assessed using nine items from a previously validated measure that asks students to respond to items on a 5-point Likert-type scale ranging from never (1) to always (5). These items have acceptable reliability [60]. Social support from teachers will be assessed using 4 items adapted from the friends and family items and using the same response scale as reported above. Questions include “… did your teachers encourage you to be physically active during recess or lunch breaks?” and “…did you teachers organise physical activity or sport for you?”

#### Behavioural strategies

Six previously validated items will be used to assess social-cognitive strategies for engaging in physical activity [60]. Students will respond to each item using a 5-point Likert-type scale ranging from never (1) to always (5). These items have acceptable test-retest reliability in adolescents [60].

#### Barriers to physical activity

Perceived barriers to physical activity will be assessed using nine items drawn from the Adolescent Physical Activity Perceived Barriers and Benefits Scales [61]. Students will respond to each item using a 4-point Likert-type scale ranging from ‘not at all true’ (1) to ‘very true’ (4). Items include “I am too busy” and “It is very hard work”. The perceived barriers scale has acceptable test-retest reliability and internal consistency [61].

#### Enjoyment

Enjoyment of physical activity will be assessed using the 16 item Physical Activity Enjoyment Scale (PACES; [62]). Students will be asked the extent to which they agree with each item (e.g. ‘I enjoy it’, ‘I dislike it’ etc.) using a 5 point Likert-type scale ranging from disagree a lot (1) to agree a lot (5). PACES has been validated for use with adolescents [62].

### Covariates

#### Anthropometry

Body mass will be measured to the nearest 0.1 kg using a calibrated electronic scale (Tanita BC-351; Tanita, Japan). Stature will be measured to the nearest 0.1 cm using SECA portable stadiometers (model 217; SECA, Germany). Waist circumference will be measured using a flexible steel tape at the narrowest point between the bottom rib and the iliac crest, in the midaxillary plane. Two measurements of body mass, stature and waist circumference will be taken and, in the event of a discrepancy over 0.1 kg or 1 cm, a third measure will be taken. The average of the two acceptable measures will be reported. Body mass index (weight/stature^2^, kg/m^2^) will also be calculated.

#### Demographics

Parents will be asked to complete a short survey that collects demographic data about the family (e.g. parent education level, employment status, marital status etc.) at baseline.

### Process evaluation feasibility measures

A range of process data will be collected to complement the outcome data collected during the study. Process data will be collected to assess the fidelity, feasibility, acceptability and appeal of RAW-PA. All process data collected will follow current process evaluation guidelines [63].

#### Feasibility/acceptability/appeal

The adolescents’ engagement with the social media intervention components will be documented (e.g. numbers reporting engagement with the resources, completion of weekly ‘missions’ or ‘challenges’, information posted by participants, Facebook ‘likes’ and comments etc.). Use of the Fitbit® throughout the 12 weeks (including missing days/syncing of data) will be collected via Fitabase (www.fitabase.com), a commercially-available platform for collecting these data from multiple users. At post-test, adolescents will complete a process evaluation questionnaire about the length of the intervention, their enjoyment and use of the different intervention components, and how they think the program could be improved. Qualitative data will be collected from adolescents (focus groups of 7–8 students per group) and school teachers (interviews) to examine the feasibility and appeal of the intervention from a participant’s and organisation’s perspective, respectively.

#### Fidelity

Data collected will include the number of text messages and emails sent by the research team, and the number of Facebook posts by the research team.

### Data analysis

The analysis of the quantitative primary and secondary outcomes will be conducted in Stata (StataCorp LP, College Station, Texas). Multilevel modelling will be used as these analyses are appropriate for the analysis of clustered data (adolescents, schools) and can handle missing data [64]. The models will assess the impact of RAW-PA (intervention, control) and adjust for potential confounders (e.g. monitor wear time, sex). Potential mediating effects will also be explored using the product-of-coefficients test of MacKinnon and colleagues [65]. Descriptive analyses will be used to examine the feasibility/acceptability/appeal of the intervention components of the study. Qualitative data from participant focus groups and teacher interviews will be analysed thematically using a mixed analysis procedure using content analysis and verbatim quotes [66].

## Discussion

Physical activity is an integral component of a healthy lifestyle. However, as only 13% of 12–14 year olds in Australia currently engage in sufficient daily physical activity to benefit their health, there is a need for efficacious strategies to increase activity levels. This is particularly true for adolescents living in socioeconomically disadvantaged areas who are an underrepresented group in physical activity interventions [14]. This is despite the fact that disadvantage is linked with declines in physical activity during the teenage years [10] and a greater risk of poor health outcomes across the life course [9]. To date, only a small number of studies have delivered interventions specifically targeting adolescents living in socioeconomically disadvantaged areas, and limited effects on physical activity levels have been observed [15, 16, 18, 20, 67].

The aim of RAW-PA, a 12 week multicomponent intervention, is to examine the effectiveness of a wearable activity tracker combined with behaviour change resources to promote physical activity in inactive adolescents attending schools in socioeconomically disadvantaged areas. It intends to capitalise on the increasing pervasiveness, appeal, and rapid uptake of wearable activity trackers, and the opportunities these devices bring to physical activity and health promotion research. RAW-PA will provide insights into how such technologies are used by adolescents, addressing an important gap in the literature to date [68]. It will identify whether combining self-monitoring via the wearable activity tracker and the accompanying resources, which are designed to help students learn and develop key behaviour change techniques, will help the adolescents to change and maintain changes in active behaviours. Moreover, utilising individual and team ‘missions’, encouraging participants to share tips for increasing activity levels, and the focus on the accumulation of physical activity every day may address potential contextual barriers often faced by those from socioeconomically disadvantaged backgrounds, such as a lack of social support, financial constraints and safety concerns [14].

An important aspect of this study is that the potential applications of the research findings, including translation and broader dissemination, have been considered. The translational aspect is often an overlooked component of behaviour change programs [69, 70]. Firstly, whilst this trial is being evaluated in urban areas of Melbourne, the online delivery of the intervention facilitates potential reach into regional and rural areas. Secondly, the intervention is being delivered via a popular social media platform, highly accessed by adolescents, and has been designed to be flexible, readily accessible and interactive. This social element addresses a key motivator for physical activity in those from socioeconomically disadvantaged areas [14]. Thirdly, as mobile phone use and internet access is ubiquitous in Australia, including in disadvantaged areas [34], this study had the potential to address potential inequities in access to structured resources often experienced by adolescents living in disadvantaged areas [71]. Since inexpensive trackers are increasingly available and costs are continuing to decrease, this may facilitate accessibility to a broader range of consumers.

This study has some limitations. Due to the multicomponent nature of the study, the effect of each individual component will not be able to be determined; though it is possible to assess the appeal and perceived effectiveness of each component via process evaluation. Second, a specific wearable activity tracker – the Fitbit® Flex - will be used. As the wearable activity tracker market is highly competitive and new devices are constantly being produced and marketed, it is possible the Flex will be superseded or become obsolete over the course of the study. However, there is no reason why the findings from this study will not be generalisable to other high quality, low cost devices that are available or will likely become available in the future.

## Conclusion

This paper has outlined the rationale and description of the RAW-PA Study for inactive adolescents attending schools located in socioeconomically disadvantaged areas. RAW-PA is an innovative physical activity intervention that combines a commercially-available activity tracker (Fitbit® Flex), accompanying app, digital resources, and a popular social media platform designed to effect behaviour change. The intervention is underpinned by participatory research principles (i.e. has been designed by adolescents for adolescents), is grounded in behaviour change theory and techniques, and incorporates a range of interactive ‘missions’ that aim to step adolescents through the behaviour change process.

## Abbreviations

CONSORT: Consolidated Standards of Reporting Trials
ICC: Intraclass correlation coefficient
MVPA: Moderate- to vigorous-intensity physical activity
RAW-PA: Raising awareness of physical activity study
RCT: Randomised controlled trial
SEIFA: The socio-economic index for areas
SES: Socio-economic status
